# Deficiency of CD44 prevents thoracic aortic dissection in a murine model

**DOI:** 10.1038/s41598-020-63824-9

**Published:** 2020-04-22

**Authors:** Omer F. Hatipoglu, Toru Miyoshi, Tomoko Yonezawa, Megumi Kondo, Naofumi Amioka, Masashi Yoshida, Satoshi Akagi, Kazufumi Nakamura, Satoshi Hirohata, Hiroshi Ito

**Affiliations:** 1 Department of Cardiovascular Medicine, Okayama University Graduate School of Medicine, Dentistry and Pharmaceutical Science, Okayama, Japan; 2 Department of Medical Technology, Graduate School of Health Sciences, Okayama University, Okayama, Japan; 3 Department of Molecular Biology and Biochemistry, Okayama University Graduate School of Medicine, Dentistry and Pharmaceutical Science, Okayama, Japan

**Keywords:** Aneurysm, Aortic diseases

## Abstract

Thoracic aortic dissection (TAD) is a life-threatening vascular disease. We showed that CD44, a widely distributed cell surface adhesion molecule, has an important role in inflammation. In this study, we examined the role of CD44 in the development of TAD. TAD was induced by the continuous infusion of β-aminopropionitrile (BAPN), a lysyl oxidase inhibitor, and angiotensin II (AngII) for 7 days in wild type (WT) mice and CD44 deficient (CD44^-/-^) mice. The incidence of TAD in CD44^-/-^ mice was significantly reduced compared with WT mice (44% and 6%, p < 0.01). Next, to evaluate the initial changes, aortic tissues at 24 hours after BAPN/AngII infusion were examined. Neutrophil accumulation into thoracic aortic adventitia in CD44^-/-^ mice was significantly decreased compared with that in WT mice (5.7 ± 0.3% and 1.6 ± 0.4%, p < 0.01). In addition, BAPN/AngII induced interleukin-6, interleukin-1β, matrix metalloproteinase-2 and matrix metalloproteinase-9 in WT mice, all of which were significantly reduced in CD44^−/−^ mice (all p < 0.01). *In vitro* transmigration of neutrophils from CD44^−/−^ mice through an endothelial monolayer was significantly decreased by 18% compared with WT mice (p < 0.01). Our findings indicate that CD44 has a critical role in TAD development in association with neutrophil infiltration into adventitia.

## Introduction

Thoracic aortic dissection (TAD) is an acute and often lethal disease characterised by disruption in the medial layer of the aortic wall resulting in the formation of a false lumen and intramural haematoma^1^. TAD is a serious medical emergency with a high mortality rate— up to 21% of patients who suffer acute aortic events (including dissection and rupture) die at home before receiving medical attention^2^. However, a large majority of TAD events occur sporadically, with no inheritance patterns^3^. Despite improvement of surgical and endovascular repair, no specific early diagnostic tool or effective therapeutic drug is available^3^. Therefore, elucidating the molecular mechanisms underlying TAD is required for the development of effective preventive and therapeutic interventions.

Medial degeneration including cystic medial necrosis is widely accepted as an important risk factor for the development of TAD^4^. However, the direct cellular and molecular mechanism that is involved in the onset of TAD has not been fully elucidated. Several studies reported that neutrophil infiltration is predominant in the acute phase of human TAD^5–7^. CD44 is a widely distributed cell surface marker and cell adhesion molecule that serves as a principle receptor for extracellular matrix components such as hyaluronan^8,9^, Under inflammatory conditions, CD44 is upregulated on inflammatory cells including neutrophils, monocytes, and lymphocytes^10^. In a murine model of atherosclerosis, CD44 was an early mediator of atherogenesis by virtue of its ability to regulate vascular gene expression in response to a proatherogenic environment^11^. CD44 then promotes the adhesion of leukocytes to endothelial cells^12^, and induces the release of inflammatory mediators from macrophages^13^. Accordingly, we hypothesised that CD44 has a critical role in TAD development in terms of neutrophil infiltration during the acute stage of TAD.

To investigate this, a mouse model in which TAD was induced by the simultaneous infusion of β-aminopropionitrile (BAPN) and angiotensin II (AngII) for 7 days was used. BAPN inhibits collagen fibre cross-linkage, and in combination with Ang II, was shown to induce aortic dissection in mice and rats. Various routes of administration including oral gavage^7,14^, subcutaneous osmotic pump infusion^15^, and subcutaneous injection^16,17^, have previously been established. In the current study, we investigated the role of CD44 in TAD induced by BAPN/AngII by comparing wild type (WT) and CD44 deficient (CD44^−/−^) mice.

## Results

### CD44 deficiency in mice decreases rates of aortic dissection

**Figure 1:**
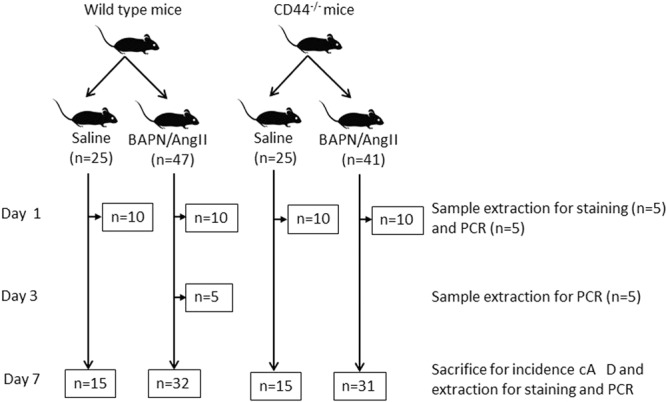
Animal protocol. To evaluate the incidence of aortic dissection, 8-week-old male WT (n = 32) or CD44^−/−^ (n = 31) mice were infused with BAPN lysyl oxidase inhibitor (100 µg/kg per minute) and AngII (1,000 ng/kg per minute) simultaneously for 7 days via osmotic minipumps. Saline infusion was used as a control for WT mice (n = 15) and CD44^−/−^ mice (n = 15). To evaluate early changes in thoracic aortae, aortic tissue was extracted from WT (n = 10) and CD44^−/−^ (n = 10) mice 1 day after BAPN/AngII or saline infusion. In addition, to analyse the expression of CD44 in thoracic aortae, tissues from WT mice were extracted from the control group 3 days after BAPN/AngII infusion (n = 5). WT: wild-type; CD44^−/−^: CD44 deficient; BAPN: β-aminopropionitrile; AngII: angiotensin II; AD:aortic dissection.

**Figure 2:**
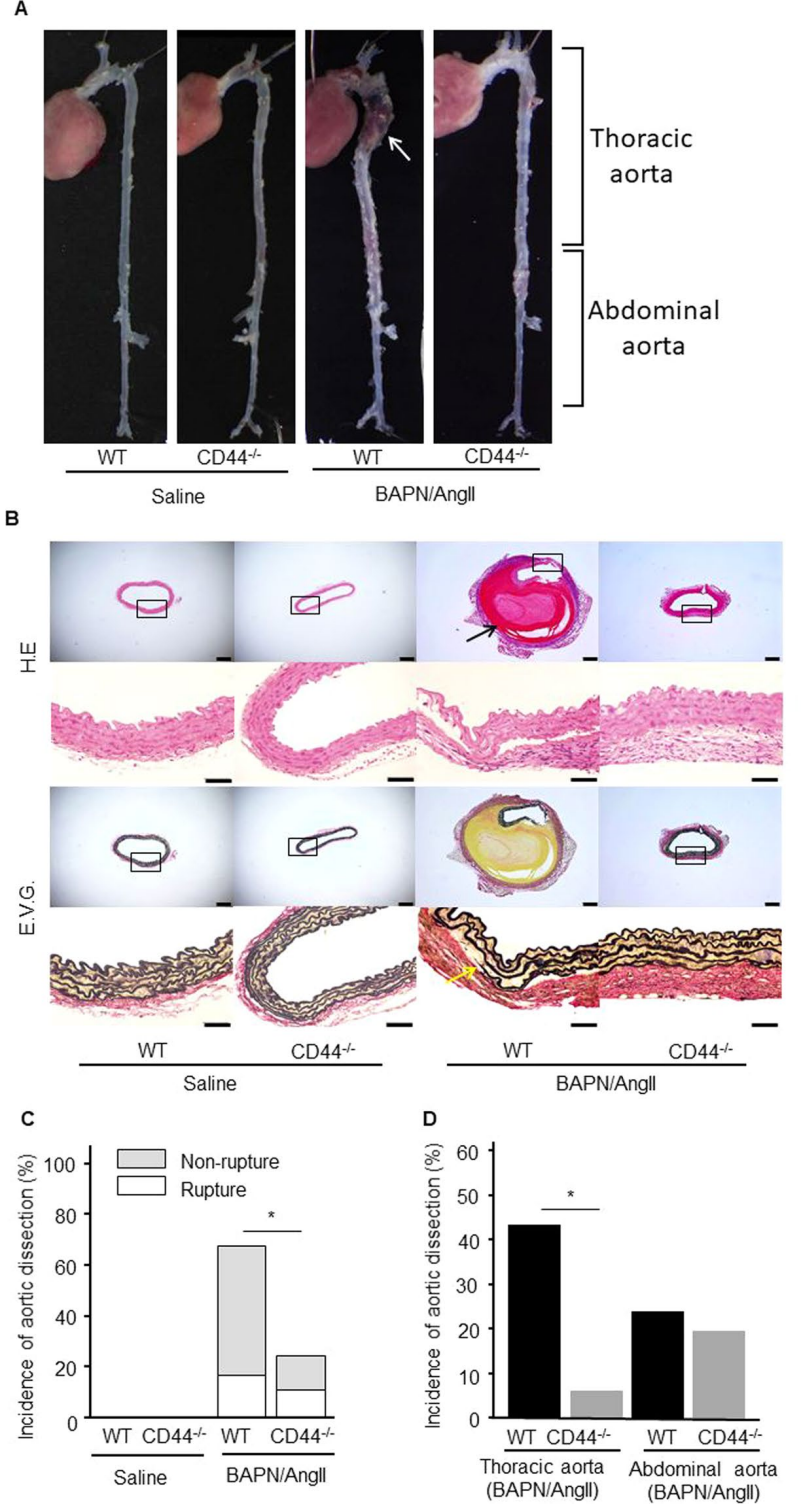
Deficiency of CD44 reduces the development of thoracic aortic dissection. (**A**) Representative photographs of aortae extracted from WT and CD44^−/−^ mice 7 days after saline or BAPN/AngII infusion. The white arrow indicates thoracic aortic dissection. (**B**) Histological analyses of thoracic aortae from WT and CD44^−/−^ mice 7 days after saline or BAPN/AngII infusion. Upper and lower panels show haematoxylin and eosin, and Elastica van Gieson staining for elastic fibres. The black arrow indicates intramural haematoma, while the yellow arrow indicates degradation of elastin. Scale bars = 200 and 50 µm. (**C**) The incidence of aortic dissection in CD44^−/−^ mice (n = 31) was significantly lower compared with WT mice (n = 32). (**D**) The decrease in the incidence of aortic dissection in CD44^−/−^ mice was observed in thoracic aortae, but not in abdominal aortae. WT: wild-type; CD44^−/−^: CD44 deficient; BAPN: β-aminopropionitrile; AngII: angiotensin II.

To investigate the effects of CD44 deficiency on the development of aortic dissection, WT and CD44^−/−^ mice were administered saline or BAPN/AngII for 7 days (Fig. 1). Saline-treated WT and CD44^−/−^ mice were used as controls. A slight increase in blood pressure after treatment with BAPN/AngII was observed in WT and CD44^−/−^ mice (Supplemental Fig. 1). Treatment with BAPN/AngII led to thoracic and abdominal aortic dissection (the presence of an intramural thrombus) and rupture. Figure 2A shows a representative image of TAD (white arrow). Histological analyses in thoracic aortae showed that intramural haematoma (black arrow) and degradation of elastin (yellow arrow) were present in the dissection area (Fig. 2B). The incidence of aortic dissection in BAPN/AngII-treated CD44^−/−^ mice was approximately 0.4 times as high as the incidence in BAPN/AngII-treated WT mice (69% and 26%, p = 0.01) (Fig. 2C). Furthermore, the incidence of TAD after BAPN/AngII treatment was significantly reduced in CD44^−/−^ mice compared with WT mice (44% and 6%, p < 0.01). However, no difference in the incidence of abdominal aortic dissection was observed (25% and 20%, p = 0.76) (Fig. 2D).

### Neutrophil migration to TAD lesions requires CD44

**Figure 3:**
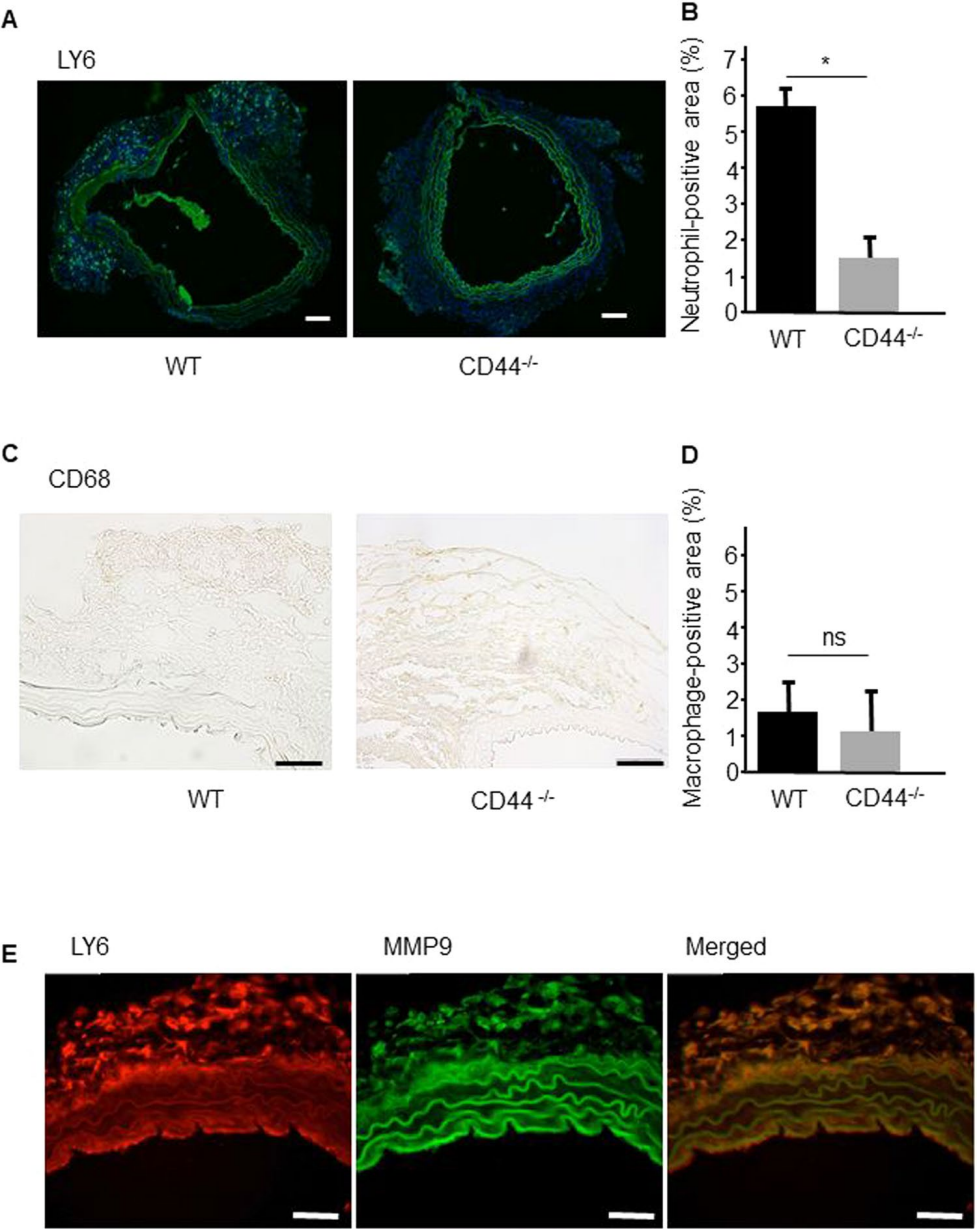
Deficiency of CD44 reduces the adventitial invasion of neutrophils into the thoracic aorta at 1 day after BAPN/AngII infusion. (**A,C**) Representative immunofluorescence staining for neutrophils (Ly6) and macrophages (CD68) in aortic sections collected from WT and CD44^−/−^ mice at 1 day after BAPN/AngII infusion. (**B,D**) Quantitative analysis shows a significant reduction of neutrophil-positive areas in aortic tissues, but not macrophage-positive areas. Results are the mean ± standard deviation of five mice. *p < 0.01 compared with WT mice. (**E**) Immunofluorescence of Ly6-positive cells (left) and MMP-9-positive cells (middle) in aortic sections of WT mice. Merged immunofluorescence showed colocalisation of Ly6- and MMP-9-positive cells. Sections are shown in the lumen below. Scale bars = 100 µm. WT: wild-type; CD44^−/−^: CD44 deficient; BAPN: β-aminopropionitrile; AngII: angiotensin II; MMP: matrix metalloproteinase.

**Figure 4:**
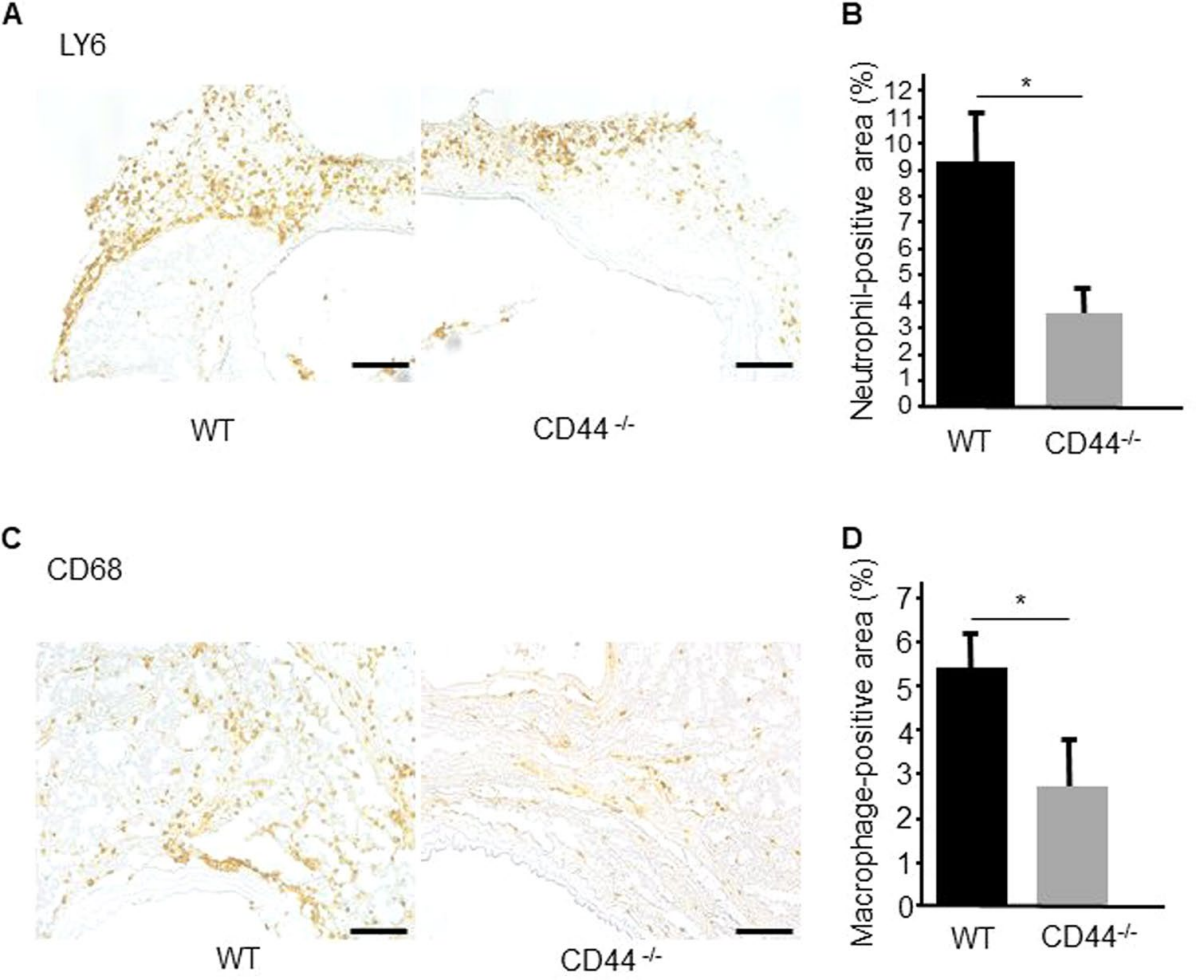
Deficiency of CD44 reduces the adventitial invasion of neutrophils and macrophages into the thoracic aorta 7 days after BAPN/AngII infusion. (**A,C**) Representative immunohistochemical staining for neutrophils (Ly6) and macrophages (CD68) in aortic sections of WT and CD44^−/−^ mice 7 days after BAPN/AngII infusion. Sections are shown in the lumen below. Scale bars = 100 µm. (**B,D**) Quantitative analysis shows a significant reduction of neutrophil- and macrophage-positive areas in aortic tissues 7 days after BAPN/AngII infusion. Results are the mean ± standard deviation of five mice. *p < 0.01 compared with WT mice. WT: wild-type; CD44^−/−^: CD44 deficient.

Several studies reported that neutrophil infiltration was predominant in the acute phase of human TAD^5–7^. Therefore, to examine the thoracic adventitial infiltration of vascular inflammatory cells during the acute stage in the TAD model, immunofluorescence staining for LY6 (neutrophils) and CD68 (macrophages) was performed 1 day after BAPN/AngII infusion. Thoracic adventitial infiltration of neutrophils in CD44^−/−^ mice was significantly lower than that in WT mice (5.7 ± 0.3% and 1.6 ± 0.4%, p < 0.01) (Figs. 3A and 3B), while a similarly very small degree of adventitial infiltration by macrophages was detected in WT and CD44^−/−^ mice (Figs. 3C and 3D). Immunofluorescence staining of the non-dissected aortae of WT mice showed that the infiltrated neutrophils in adventitia predominantly expressed matrix metalloproteinase (MMP)−9 (Fig. 3E). In addition, on day 7 after BAPN/AngII infusion, immunohistochemical staining showed that the numbers of neutrophils and macrophages in CD44^−/−^ mice remained significantly lower than in WT mice (9.6 ± 1.7% and 3.2 ± 1.3%, p < 0.01, and 5.5 ± 0.9% and 2.7 ± 0.8%, p < 0.01, respectively) (Fig. 4A–D).

### CD44 deficiency in mice reduces the expression of proinflammatory cytokines

**Figure 5:**
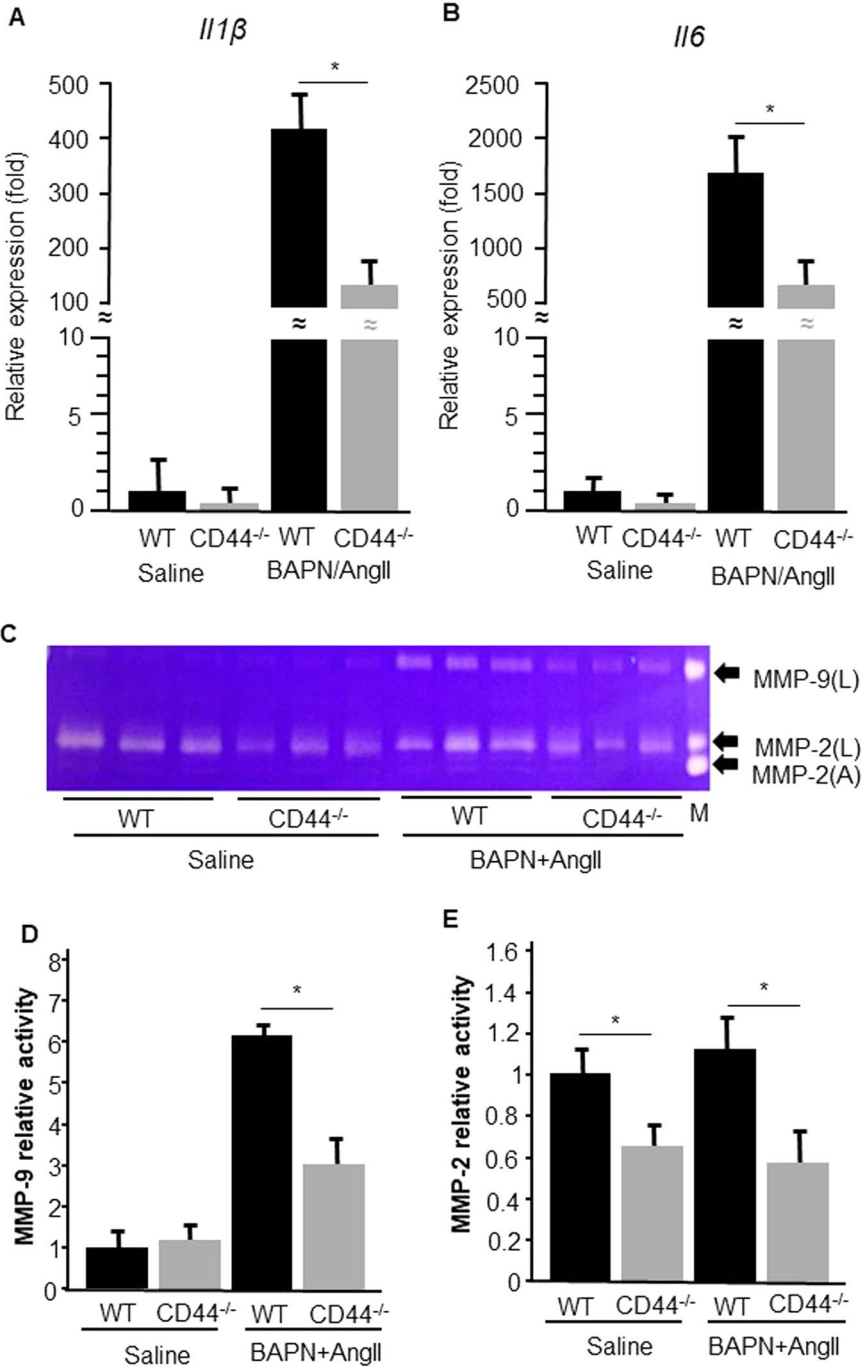
Inflammatory cytokines and MMP-9 activity are reduced in CD44^−/−^ mice. (**A,B**) Total RNA was extracted from the thoracic aortae of WT and CD44^−/−^ mice infused with saline or BAPN/AngII for 24 hours, and mRNA expressions of *Il1b* and *Il6* were analysed by quantitative polymerase chain reaction (n = 5 for each). (**C**) MMP-2 and MMP-9 activities in aortic aortae from WT and CD44^−/−^ mice infused with saline or BAPN/AngII for 24 hours were analysed by gelatin zymography. The latent (L) and active (A) forms of MMP-2 and MMP-9 are indicated by arrows. These zymograms are representative results from three independent experiments. (**D,E**) The relative changes in MMP-2 and MMP-9 activities were quantified using ImageJ software (n = 3). Results are the mean ± standard deviation. M = MMP marker. *p < 0.01. WT: wild-type; CD44^−/−^: CD44 deficient; BAPN: β-aminopropionitrile; AngII: angiotensin II; MMP: matrix metalloproteinase.

To investigate the effect of CD44 deficiency on inflammatory cytokines *in vivo*, cytokine mRNA expression in thoracic aortae isolated from WT and CD44^−/−^ mice was examined. Quantitative polymerase chain reaction (qPCR) showed that the gene expressions of interleukin (IL)−1β and IL-6 in each group treated with BAPN/Ang II for 1 day were significantly greater than those in each group treated with saline (all p < 0.01). However, gene expressions of IL-1β and IL-6 in CD44^−/−^ mice treated with BAPN/Ang II for 1 day were significantly lower than those in WT mice (Figs. 5A and 5B). We examined the change in CD44 expression in thoracic aortae after BAPN/AngII infusion and found that CD44 expression in WT mice was significantly increased from day 1 to day 7 after BAPN/AngII infusion (all p < 0.01) (Supplemental Fig. 2).

### CD44 deficiency attenuates BAPN/AngII-induced MMP-9 activation

We determined MMP activity in the thoracic aortae of WT and CD44^−/−^ mice after infusion by BAPN/Ang II for 1 day. Results of gelatin zymography demonstrated that BAPN/Ang II infusion upregulated MMP-9 levels (Fig. 5C). Furthermore, MMP-9 levels after BAPN/Ang II infusion for 1 day were significantly increased in WT aortae compared with CD44^−/−^ aortae (p < 0.01, Fig. 5D). A significant increase in MMP-2 level in WT mice and CD44^−/−^ mice after BAPN/Ang II infusion was not observed, whereas MMP-2 levels in WT mice were significantly higher than in CD44^−/−^ mice after the administration of saline (p < 0.01) or BAPN/Ang II infusion (p = 0.04) (Fig. 5E).

### Migratory capabilities of CD44-/–derived neutrophils are lower than those of WT-derived neutrophils

**Figure 6:**
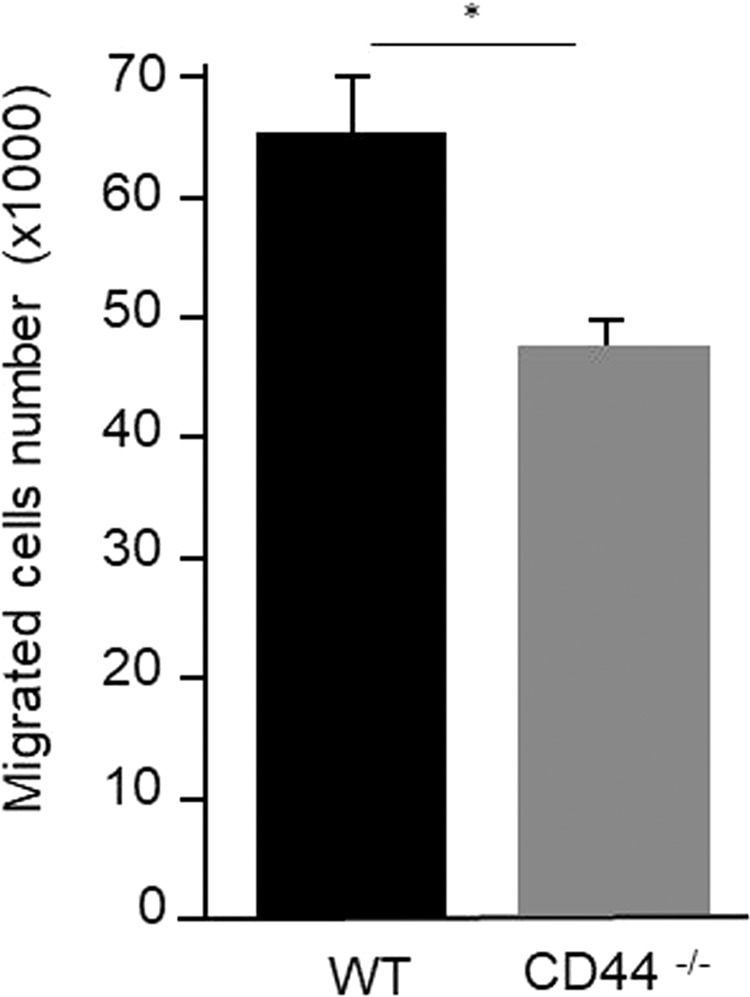
Capacity of neutrophils derived from CD44^−/−^ and WT mice to infiltrate through endothelial cell layers. The number of neutrophils that transmigrated through endothelial cells layer was assessed with a Boyden Chamber assay. Neutrophils from CD44^−/−^ mice had a significantly lower infiltration capability than those from WT mice. *p < 0.01. WT: wild-type; CD44^−/−^: CD44 deficient.

Neutrophils were isolated from bone marrow by fluorescence activated cell sorting. After negative immunomagnetic separation, the isolated cell purity was > 85% neutrophils (data not shown). Neutrophil transmigration through the endothelial cell monolayer is a key process in the inflammatory response. To investigate whether CD44 plays an active role in neutrophil transmigration through vascular endothelium, transwell assays with WT and CD44^−/−^ mouse-derived neutrophils were performed. The transmigration of neutrophils from CD44^−/−^ mice was significantly decreased compared with WT-derived neutrophils (p < 0.01) (Fig. 6).

## Discussion

In this study, we provide evidence that CD44 has an important role in TAD formation. Using a BAPN/Ang II-treated murine model, we showed that CD44 deficiency significantly prevented aortic dissection in thoracic, but not in abdominal, segments. CD44 deficiency reduced neutrophil infiltration into adventitia. *In vitro* experiments found that CD44 deficiency reduced the migratory capability of neutrophils. Together, our results suggest that CD44 has a critical role in the development of TAD.

Previous studies show that neutrophil infiltration is predominant in the acute phase of human TAD^5–7^. In a murine model of thoracic aortic dissection, neutrophils were shown to be an important mediator in the initiation of TAD, as the antibody-mediated depletion of neutrophils attenuated TAD formation^7^. In the current study, substantial differences in neutrophils, but not monocytes, infiltrating the adventitia between WT mice and CD44^−/−^ mice were observed at the early stage of disease. We also demonstrated that MMP-9 was expressed by adventitial neutrophils, but not by neutrophils in the middle layer, in non-dissected aortae. Our results are in agreement with previous findings that neutrophil infiltration is a critical step preceding TAD.

Regarding the increase in neutrophils in the adventitia 1 day after BAPN/AngII infusion, our cell culture experiments demonstrated that the migration capability of CD44^−/−^ mouse-derived neutrophils through the endothelial cell layer was significantly lower than that of WT mouse-derived neutrophils. The mechanisms of neutrophil adhesion to endothelium are partially understood^12,18^, CD44 was shown to be critical for the polarisation and migration of mouse neutrophils and CD44^−/−^ neutrophils displayed a slow migration and speed, and reduced activation^19^. Thus, CD44 has an important role in neutrophil adhesion. The lower migration capability of neutrophils in CD44^−/−^ mice may explain the difference between CD44^−/−^ and WT mice in the number of neutrophils infiltrated into adventitia at the early stage of disease.

As CD44 is present on many types of cells, the reduced neutrophil infiltration in adventitia in CD44^−/−^ mice may also be a result of the lower inflammatory status of the medial layer of the aorta. In fact, the current study showed that the mRNA expressions of IL-6 and IL-1β in thoracic aortic tissues at 1 day after BAPN/AngII infusion were greater in WT mice than in CD44^−/−^ mice. This may be due to the regulatory effect of CD44 on inflammation via the hyaluronan-CD44 pathway^20^. CD44 is a major receptor of hyaluronan, which is abundantly present in aortic tissues^8,9^ Hyaluronan exists over a broad range of different molecular weights that have contrasting effects on cell behaviour. Low molecular weight hyaluronan tends to be proinflammatory, whereas high molecular weight hyaluronan tends to have anti-inflammatory properties^21^. We previously reported that low molecular weight hyaluronan induced IL-6 and monocyte chemoattractant protein-1 production in peripheral blood mononuclear cells^13^. In our model, inflammation induced by AngII might cause the subsequent breakdown of hyaluronan in aortic tissues, which might augment inflammation in the aorta^20^. The role of the hyaluronan-CD44 pathway in TAD requires further clarification in future studies.

Under inflammatory conditions, CD44 is upregulated and functionally activated on vascular endothelial, smooth muscle, and inflammatory cells^20^. Krettek *et al*. also reported the increased expression of CD44 on macrophages, smooth muscle cells, endothelial cells, and CD4-positive T cells in a human abdominal aortic aneurysm^22^. Thus, increased CD44 expression on various cells might orchestrate inflammation in aortic tissues.

Clinically, TAD is an aggressive vascular disease that requires early diagnosis and treatment. The preliminary screening of patients with suspected acute aortic dissection in an emergency room is done using transthoracic echocardiography, computed tomography angiography, or both. However, these methods cannot provide early detection of dissection before intimal tear. Thus, the early detection of TAD requires the development of molecular imaging that combines the early characteristic pathological processes and classic imaging^23^. For example, type IV collagen, a major component of the sub-endothelial basement membrane, was investigated as a target of molecular imaging^24^. As CD44 acts as a receptor for hyaluronan, hyaluronan-based nanocarriers can be used as CD44-targeted molecules. Several anti-cancer drugs have been incorporated in the inner hydrophobic part of hyaluronan-nanocarriers and tested for their therapeutic efficacy^25^. In the future, CD44-targeted molecules could be developed to detect early changes indicating a propensity for TAD.

In conclusion, the current study provides direct evidence that CD44 has a role in TAD. Further studies are necessary to clarify the precise mechanism whereby CD44 is detrimental in TAD.

## Methods

### Murine model of BAPN/Ang II-induced aortic dissection

Eight-week-old male WT (C57BL/6 J) and CD44^−/−^ mice were purchased from The Jackson Laboratory (Bar Harbor, ME). The room temperature was maintained at 22 ± 2 °C, with a 12-h/12-h day/night cycle and relative humidity of 50 ± 10%. Food and water were available *ad libitum*. Figure 1 shows the animal protocol of this study. For the induction of TAD, WT (n = 32) or CD44^−/−^ (n = 31) mice were infused with BAPN, a lysyl oxidase inhibitor (100 µg/kg per minute) and AngII (1,000 ng/kg per minute) simultaneously for 7 days via osmotic minipumps (Model 2001; DURECT Corporation, Cupertino, CA). Saline infusion was used as the control in WT (n = 25) and CD44^−/−^ (n = 15) mice. BAPN was purchased from Sigma (St. Louis, MO) and AngII was purchased from Bachem (Saint Helens, UK). All animal experiments were performed in accordance with protocols approved by the Animal Research Committee of Okayama University based on the Animal Research: Reporting of *In Vivo* Experiments guidelines.

### Histological analysis

The aortic tissues from different groups of mice were fixed with 10% neutral buffered formalin, embedded in paraffin, cut into 5-µm-thick sections using a microtome (Microm HM400), and placed on an adhesive glass slide (Platinum Pro; Matsunami, Osaka, Japan). The slides were air-dried at room temperature for 2 hours. Haematoxylin and eosin, and Elastica van Gieson staining were performed using standard protocols. For immunostaining, the tissue sections were blocked with 5% bovine serum albumin in phosphate-buffered saline for 1 hour at room temperature, and then incubated overnight at 4 °C with a rat anti-mouse LY-6 antibody (BD Pharmingen, 550291, 1:700), rabbit anti-MMP-9 antibody (Millipore, AB19016, 1:500), and rat anti-mouse CD68 (MCA1957, Bio-Rad, 1:700). Sections were then incubated for 60 minutes at room temperature with the following secondary antibodies: goat anti-rat IgG Alexa Fluor 488 (Thermo Fisher Scientific, A11006, 1:1500), chicken anti-rabbit IgG Alexa Fluor 488 (Thermo Fisher Scientific, A21441, 1:1500), goat anti-rat Alexa Fluor 594 (Thermo Fisher Scientific, A11007, 1:1500), and biotinylated goat anti-rat Ig secondary antibody (BD Pharmingen, 559286, 1:1500). A Vector ABC Elite kit and DAB Impact solution (Vector Labs, Burlingame CA) were used for diaminobenzidine staining. The sections were examined with a Biozero BZ-X700 microscope (Keyence, Japan) at the Central Research Laboratory, Okayama University Medical School.

### Quantitative real-time PCR

Total RNA was extracted with TRIzol reagent (Invitrogen, Carlsbad, CA) in accordance with the manufacturer’s protocol. RNA was quantified using Implen’s Nanophotometer (Munich, Germany). Contaminating DNA was digested with DNase I (Takara, Tokyo, Japan). cDNA was synthesised using the Prime Script RT reagent kit (Takara) following the manufacturer’s instructions. Real-time qPCR was performed using the TaqMan Universal PCR Master Mix (Applied Biosystems, CA) and an Applied Biosystems 7500 Fast Real-Time PCR System (Applied Biosystems)^26^. All mRNA-specific labelled primers were purchased from Applied Biosystems and detected cDNA from the following genes: *Il1b* (Mm00434228_m1), *Il6* (Mm00446190_m1), and *Cd44* (Mm01277161_m1). The *Gapdh* (Mm99999915_g1) gene was used as an internal control and the data were analysed using the 2^−ΔΔCt^ method.

### Gelatin zymography

The enzymatic activities of MMP-2 and MMP-9 were analysed using a gelatine zymogram kit in accordance with the manufacturer’s protocol (PMC-AK47-COS; Cosmo Bio, Tokyo, Japan). Briefly, WT mice and CD44^−/−^ mice were treated with BAPN/Ang II for 24 hours. Protein was isolated from thoracic aortae, and the total protein concentrations were determined using the Bradford assay (Bio-Rad Laboratories, Hercules, CA). Total protein (10 µg) was mixed with non-reducing Laemmli sample buffer and separated using 10% sodium dodecyl sulfate - polyacrylamide gel electrophoresis that included 1 mg/ml gelatine without prior heating. After electrophoresis, the gel was washed with 2.5% Triton X-100 solution for 30 minutes to remove all sodium dodecyl sulfate, then incubated in 50 mM Tris-HCl, 5 mM CaCl_2_, and 1 µM ZnCl_2_ for 16 hours at 37 °C. Following incubation, the gels were stained with 0.05% Coomassie brilliant blue R-250 for 30 minutes at room temperature and then destained with wash buffer and photographed. Latent and active forms of MMP-2 and MMP-9 were visualised as colourless bands on a blue background. ImageJ software was used to determine the colour density of the band formation area.

### Cell culture

Human umbilical vein endothelial cells were grown in EBM-2 medium supplemented with EGM-2 SingleQuots (Lonza, Walkersville, MD). Murine neutrophils were grown in RPMI medium (Gibco, Langley, OK) containing 10% foetal bovine serum (Gibco) and penicillin and streptomycin (Wako, Tokyo, Japan).

### Isolation of bone marrow-derived neutrophils by negative depletion

First, 8–10-week-old mice were euthanased and bones were pooled from 4 to 5 mice. The epiphyses of the bones were removed, and bone marrow cells were flushed into a 50 ml conical tube through a 100-µm cell strainer (Thermo Scientific, Worcester, MA). Tissue fragments were removed using a 25-gauge needle and a 10 ml syringe filled with RPMI medium. After centrifugation (500 ×g/5 minutes/RT), red blood cells were lysed with RBC Lysis Buffer (eBioscience, San Diego, CA). Neutrophils were isolated using MojoSort Mouse Neutrophil Isolation Kit (BioLegend, San Diego, CA). Cells were resuspended in 1 ml phosphate-buffered saline (PBS) with 10 µl of MojoSort Biotin-Antibody Cocktail and the suspension was constantly turned in a rotating shaker at 4 °C for 30 minutes. Then the supernatant was removed (500 ×g/5 minutes/RT). After washing twice with PBS, the cell pellet was suspended in 1 ml PBS with 10 µl MojoSort Streptavidin Nanobeads, then shaken at 4 °C for 30 minutes and the supernatant removed (500 ×g/5 minutes/RT). Cells were resuspended in 1 ml PBS and the tube was attached to a magnet on the tube hinge side to obtain the bone marrow-derived neutrophils that did not attach to the beads. Supernatant was aspirated carefully from the tube. The obtained cells were then washed once with 10 ml RPMI medium. Neutrophils were cultivated overnight in RPMI medium containing 10% foetal calf serum and penicillin and streptomycin. After isolating neutrophils, they were stained with an anti-CD11b FITC/anti-LY6G PE mixture and analysed for double positive cells by flow cytometry.

### Leukocyte transendothelial migration experiments

Quantitative transendothelial migration was assayed using the Cytoselect Leukocyte Transmigration Assay kit (6.5-mm diameter, 8-µm pore size, CBA-101, Cell Biolabs, Inc., San Diego, CA) following the manufacturer’s instructions. Briefly, human umbilical vein endothelial cells were plated at confluent density (7.5×10^5^ cells/cm^2^) on transwell filter inserts. After 2 days, cells were activated with 5 ng/ml of IL-1 overnight. CD44^−/−^ and WT mice-derived neutrophils were placed in the upper transwell chambers (1×10^6^ cells) and allowed to transmigrate for 18 hours at 37 °C, after which the medium from the bottom well was collected, and transmigrated cells were lysed and quantified with a Flex Station 3 plate reader (Molecular Device, Sunnyvale, CA) using 485 and 535 nm excitation and emission filters, respectively.

### Statistical analysis

All quantitative data are presented as the means ± standard deviation. Statistical analyses were performed with SPSS software (version 24; SPSS, Inc., Chicago, IL). To compare the incidence of aortic dissection between two groups, Fisher’s exact test was performed. To compare continuous variables among four groups, one-way analysis of variance with post-hoc Tukey-Kramer testing was performed. To compare heart rate and blood pressures between two groups, two-way repeated analysis of variance was used. All data passed normality of distribution. A *P* value <0.05 was considered statistically significant.

## Supplementary information

Supplementary Information.
