# Supplementary material for: Deficiency of CD44 prevents thoracic aortic dissection in a murine model
Source: Sci Rep. 2020 Apr 22;10:6869. doi: 10.1038/s41598-020-63824-9 (PMC7176701; doi:10.1038/s41598-020-63824-9)
Supplement: Supplementary file 1 — Supplementary Information. [file 41598_2020_63824_MOESM1_ESM.docx]

**Supplemental File**

**Deficiency of CD44 prevents thoracic aortic dissection in a murine model**

Omer F. Hatipoglu^1,2^, Toru Miyoshi^1^*, Tomoko Yonezawa^3^, Megumi Kondo^1^, Naofumi Amioka^1^, Masashi Yoshida^1^, Satoshi Akagi^1^, Kazufumi Nakamura^1^, Satoshi Hirohata^2^, Hiroshi Ito^1^

1. Department of Cardiovascular Medicine, Okayama University Graduate School of Medicine, Dentistry and Pharmaceutical Science, Okayama, Japan
2. Department of Medical Technology, Graduate School of Health Sciences, Okayama University, Okayama, Japan.
3. Department of Molecular Biology and Biochemistry, Okayama University Graduate School of Medicine, Dentistry and Pharmaceutical Science, Okayama, Japan

**Methods**

**Blood pressure and heart rate measurements**

Systolic blood pressure and heart rate were measured by a noninvasive computerized tail-cuff system (Muromachi Kikai Co., Ltd., Tokyo, Japan). Five measurements were obtained from each non-anesthetized mouse (five mice per group). To compare heart rate and blood pressure between two groups, two-way repeated analysis of variance was used.

**
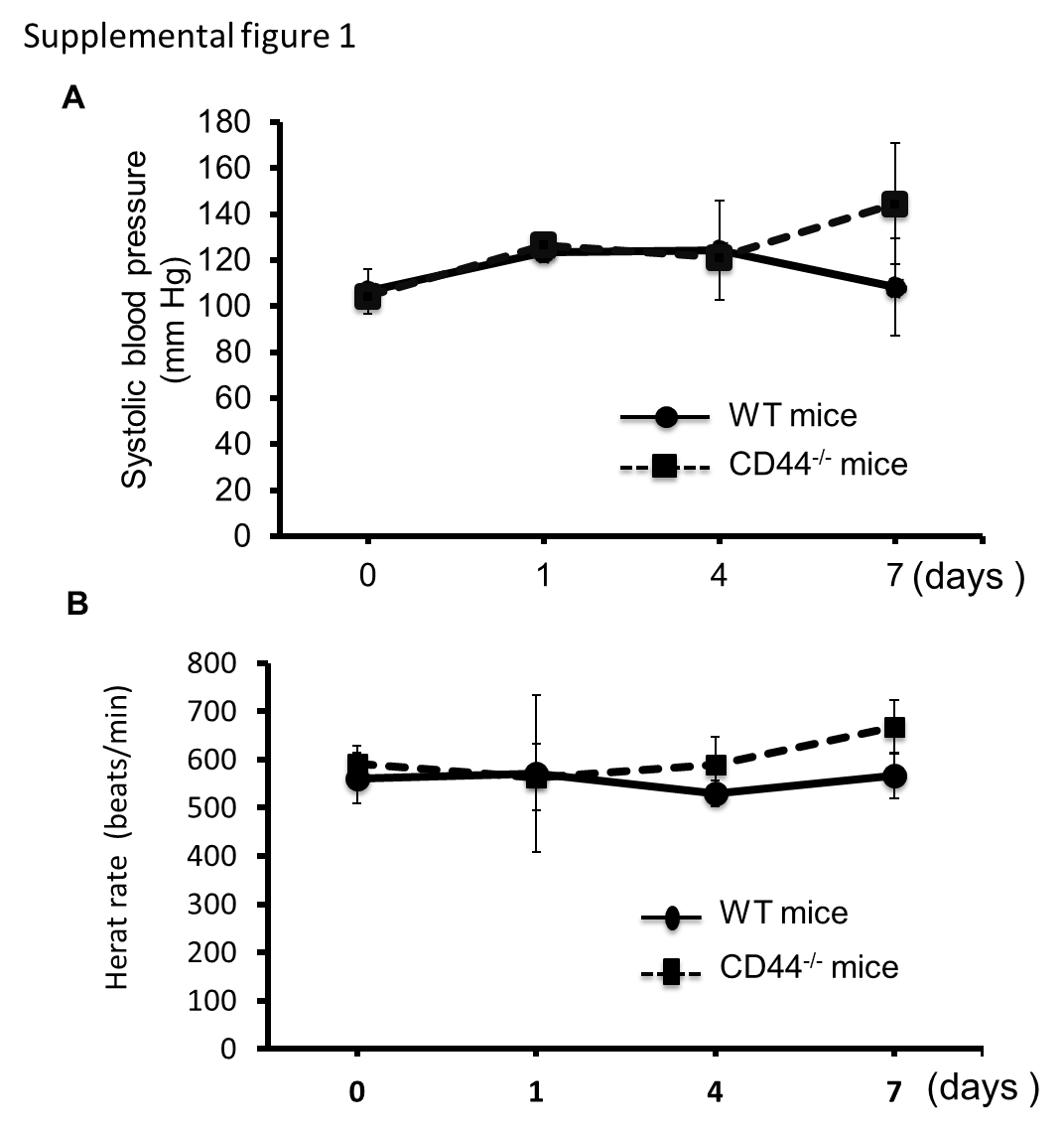
**

**Supplemental Figure 1.** Change in blood pressure and heart rate. (A) Systolic blood pressure and (B) heart rate in WT and CD44^-/-^ mice were serially measured with a tail-cuff for 7 days after BAPN/AngII administration. n=5 for each group.

**
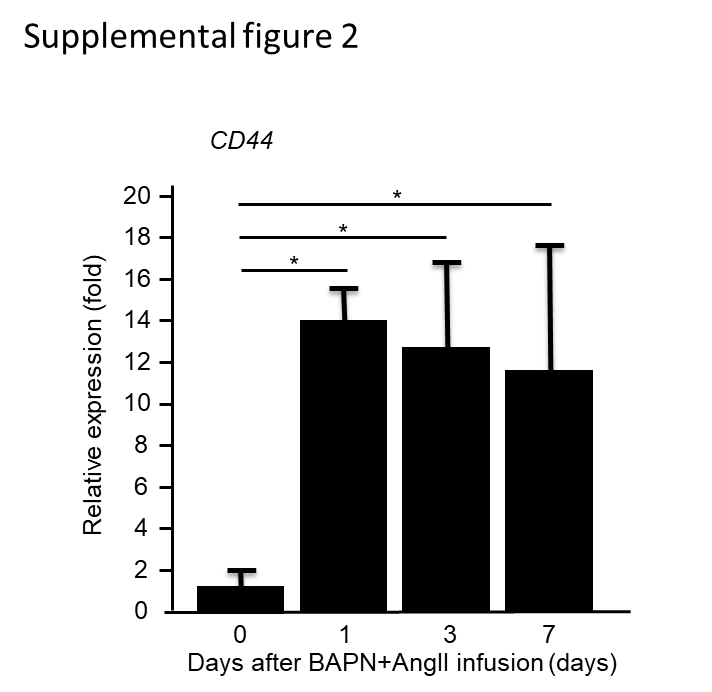
**

**Supplemental Figure 2.** Change in CD44 expression in thoracic aorta.

Total RNA was extracted from the thoracic aorta of WT mice before and after BAPN/AngII administration. mRNA expression of CD44 was analysed by real-time PCR (n=4).
